# Metformin Mitigated Obesity-Driven Cancer Aggressiveness in Tumor-Bearing Mice

**DOI:** 10.3390/ijms23169134

**Published:** 2022-08-15

**Authors:** Chun-Jung Chen, Chih-Cheng Wu, Cheng-Yi Chang, Jian-Ri Li, Yen-Chuan Ou, Wen-Ying Chen, Su-Lan Liao, Jiaan-Der Wang

**Affiliations:** 1 Department of Medical Research, Taichung Veterans General Hospital, Taichung City 407, Taiwan; 2 Department of Medical Laboratory Science and Biotechnology, China Medical University, Taichung City 404, Taiwan; 3 Department of Anesthesiology, Taichung Veterans General Hospital, Taichung City 407, Taiwan; 4 Department of Financial Engineering, Providence University, Taichung City 433, Taiwan; 5 Department of Data Science and Big Data Analytics, Providence University, Taichung City 433, Taiwan; 6 Department of Surgery, Feng Yuan Hospital, Taichung City 420, Taiwan; 7 Department of Veterinary Medicine, National Chung Hsing University, Taichung City 402, Taiwan; 8 Division of Urology, Taichung Veterans General Hospital, Taichung City 407, Taiwan; 9 Division of Urology, Tungs’ Taichung Metro Harbor Hospital, Taichung City 435, Taiwan; 10 Children’s Medical Center, Taichung Veterans General Hospital, Taichung City 407, Taiwan; 11 Department of Industrial Engineering and Enterprise Information, Tunghai University, Taichung City 407, Taiwan

**Keywords:** salignancy, metabolism, metformin, neutrophils, neutrophil extracellular traps, obesity

## Abstract

Metformin may offer benefits to certain cancer populations experiencing metabolic abnormalities. To extend the anticancer studies of metformin, a tumor model was established through the implantation of murine Lewis Lung Carcinoma (LLC) cells to Normal Diet (ND)-fed and High-Fat Diet (HFD)-fed C57BL/6 mice. The HFD-fed mice displayed metabolic and pro-inflammatory alterations together with accompanying aggressive tumor growth. Metformin mitigated tumor growth in HFD-fed mice, paralleled by reductions in circulating glucose, insulin, soluble P-selectin, TGF-β1 and High Mobility Group Box-1 (HMGB1), as well as tumor expression of cell proliferation, aerobic glycolysis, glutaminolysis, platelets and neutrophils molecules. The suppressive effects of metformin on cell proliferation, migration and oncogenic signaling molecules were confirmed in cell study. Moreover, tumor-bearing HFD-fed mice had higher contents of circulating and tumor immunopositivity of Neutrophil Extracellular Traps (NETs)-associated molecules, with a suppressive effect from metformin. Data taken from neutrophil studies confirmed the inhibitory effect that metformin has on NET formation induced by HMGB1. Furthermore, HMGB1 was identified as a promoting molecule to boost the transition process towards NETs. The current study shows that metabolic, pro-inflammatory and NET alterations appear to play roles in the obesity-driven aggressiveness of cancer, while also representing candidate targets for anticancer potential of metformin.

## 1. Introduction

The prevalence and incidence of being overweight or obese are increasing rapidly worldwide. Beyond their direct negative effects on human health, obesity certainly leads to a steep rise in related health complications and poor outcomes. Type 2 diabetes mellitus and cancer are common health complications surrounding obesity. Additionally, an obese population with accompanying type 2 diabetes mellitus is particularly threatened by cancer, being predisposed to suffering from its incidence and progression [1,2]. The involvement of obesity-related alterations in cancer and possible treatment resistance is multifactorial; therefore, obesity targeting represents an emerging strategy for the prevention of obesity-driven malignant progression [3]. To meet such clinical needs, the elucidation of obesity-related alterations and the identification of targetable candidates are of great importance.

Obesity is a metabolic disorder concerning a dysregulated metabolism in glucose, lipids and macromolecules. Hyperglycemia, hyperinsulinemia, dyslipidemia and low-grade inflammation, along with an increase in adipokines, total White Blood Cells (WBCs), neutrophils and Neutrophil Extracellular Traps (NETs), as well as platelet activation, are common biochemical hallmarks of obesity [4,5,6]. Such obesity-related alterations and circulating factors have been implicated in the progression of cancer through proliferation, shielding, escape from immune surveillance, distant metastasis, tumor-prone vasculature and microenvironment [3,7,8,9]. Therefore, obesity targeting treatment and intervention centered on obesity-related alterations are both potential strategies for reversing the cancer-promoting effects of obesity.

Obesity-oriented therapeutic compounds such as Sodium-Glucose Cotransporter-2 (SGLT2), statins and metformin all show tumor growth inhibitory effects, and reverse obesity-driven cancer aggressiveness [10,11,12]. Beyond its glucose-lowering and anti-diabetes effects, epidemiological, clinical and experimental evidence suggest additional therapeutic applications of metformin with regard to anticancer potential [13,14,15]. Preclinical studies have uncovered the anticancer potential of metformin for use in monotherapy, adjuvant treatment and combinatory treatment, while revealing several oncogenic molecules pivotal to its action. Reduction in circulating insulin and activation of 5′-AMP-Activated Protein Kinase (AMPK) are two reported anticancer mechanisms of metformin [16,17]. Moreover, Nuclear Factor Erythroid 2-Related Factor 2 (Nrf2), Yes-Associated Protein (YAP), Hypoxia-Inducible Factor-1α (HIF-1α), High-Mobility Group Box 1 (HMGB1), Transforming Growth Factor-β1 (TGF-β1), glycolysis, glutamine metabolism and antitumor immunity have all been further highlighted as functional targets in the anticancer actions of metformin [14,18,19,20,21,22,23]. Regarding the pro-malignant effects of platelets and neutrophils, metformin inhibits both the release of NETs and hyper-activity of platelets [24,25,26]. Despite the aforementioned achievements, the anticancer mechanisms surrounding metformin are still not completely understood.

Studies have been performed showing the cancer-preventive effects of metformin in tumor-bearing rodents with obese or diabetic backgrounds involving targeting insulin, AMPK, fat metabolism, antiapoptotic molecules, Signal Transducer and Activator of Transcription 3 (Stat3) and Extracellular Signal-Regulated Kinase (ERK) [27,28,29,30,31]. Previously, we reported on the anticancer effects of antiplatelet drugs, such as aspirin and dipyridamole, in a syngeneic tumor model produced by murine Lewis Lung Carcinoma (LLC) cells and C57BL/6 mice [32,33]. To extend the scope of our metformin studies, in the current study, we aimed to investigate its anticancer effects on obesity-driven cancer development while focusing on tissue oncogenic pathways and blood-borne cancer-promoting mechanisms by taking advantage of the syngeneic tumor model.

## 2. Results

### 2.1. Metformin Mitigated Tumor Growth in Tumor-Bearing Obese Mice

**Figure 1:**
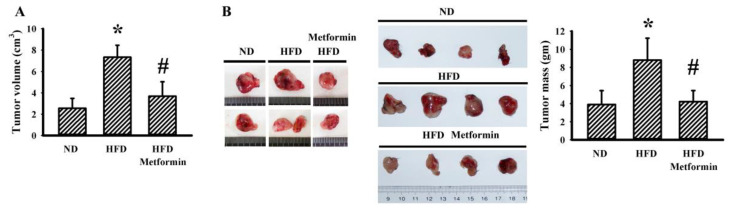
Metformin mitigated tumor growth in obese mice. ND-fed lean and HFD-fed obese mice were subcutaneously inoculated with LLC cells over a course of three weeks. Three days after cell implantation, ND-fed lean mice were treated daily with saline vehicle through intraperitoneal injection (ND). HFD-fed obese mice were separated into two groups receiving daily intraperitoneal injections of a saline vehicle (HFD) or metformin (50 mg/kg, HFD/Metformin) for the final 18 days. The tumor volumes were measured (**A**) and the resected tumor tissues were weighed (**B**). Representative resected tumors are shown (**B**). * *p* < 0.05 vs. ND and # *p* < 0.05 vs. HFD, n = 6.

**Figure 2:**
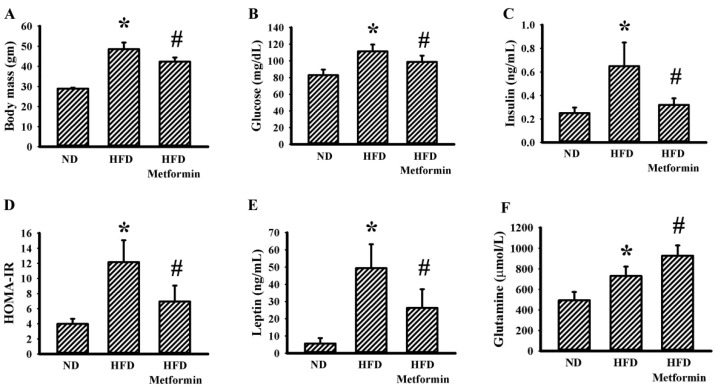
Metformin mitigated metabolic alterations in obese mice. ND-fed lean and HFD-fed obese mice were subcutaneously inoculated with LLC cells over a course of three weeks. Three days after cell implantation, ND-fed lean mice were treated daily with saline vehicle through intraperitoneal injection (ND). HFD-fed obese mice were separated into two groups receiving daily intraperitoneal injections of a saline vehicle (HFD) or metformin (50 mg/kg, HFD/Metformin) for the final 18 days. Body mass (**A**), fasting glucose (**B**), fasting insulin (**C**), HOMA-IR (**D**), plasma leptin (**E**) and plasma glutamine (**F**) were all determined. * *p* < 0.05 vs. ND and # *p* < 0.05 vs. HFD, n = 6.

**Figure 3:**
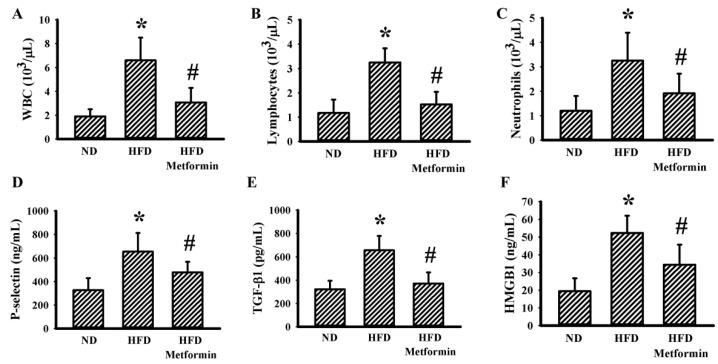
Metformin mitigated inflammatory alterations in obese mice. ND-fed lean and HFD-fed obese mice were subcutaneously inoculated with LLC cells over a course of three weeks. Three days after cell implantation, ND-fed lean mice were treated daily with saline vehicle through intraperitoneal injection (ND). HFD-fed obese mice were separated into two groups receiving daily intraperitoneal injections of a saline vehicle (HFD) or metformin (50 mg/kg, HFD/Metformin) for the final 18 days. The levels of WBCs (**A**), lymphocytes (**B**), neutrophils (**C**), soluble P-selectin (**D**), TGF-β1 (**E**) and HMGB1 (**F**) in blood samples were all determined. * *p* < 0.05 vs. ND and # *p* < 0.05 vs. HFD, n = 6.

Ectopic tumor growth was produced via the implantation of LLC cells into male C57BL/6 mice that were pre-fed with a Normal Diet (ND) or High-Fat Diet (HFD) over a period of 10 weeks. Within a period of 3 weeks, tumors had grown more aggressively in the HFD-fed obese mice than in the ND-fed lean mice, as evidenced by an increase in both tumor volume (Figure 1A) and tumor mass (Figure 1B). Metformin displayed a suppressive effect on both tumor volume (Figure 1A) and tumor mass (Figure 1B) in HFD-fed mice. Regarding obesity associated metabolic and pro-inflammatory alterations, tumor-bearing HFD obese mice had a higher body mass (Figure 2A), fasting glucose (Figure 2B), fasting insulin (Figure 2C) and Homeostasis Model Assessment (HOMA) insulin resistance (HOMA-IR) (Figure 2D), as well as circulating leptins (Figure 2E), glutamine (Figure 2F), WBCs (Figure 3A), lymphocytes (Figure 3B), neutrophils (Figure 3C), soluble P-selectin (Figure 3D), TGF-β1 (Figure 3E) and HMGB1 (Figure 3F). Metformin mitigated all changes in tumor-bearing HFD-fed obese mice, with the exception of glutamine, where metformin caused an additional elevation of glutamine (Figure 2 and Figure 3). These findings indicate that the tumor growth inhibitory effect of metformin is accompanied by improved metabolic and pro-inflammatory alterations in tumor-bearing HFD-fed obese mice.

### 2.2. Metformin Mitigated Signaling Molecule Expression in Tumor Tissues

**Figure 4:**
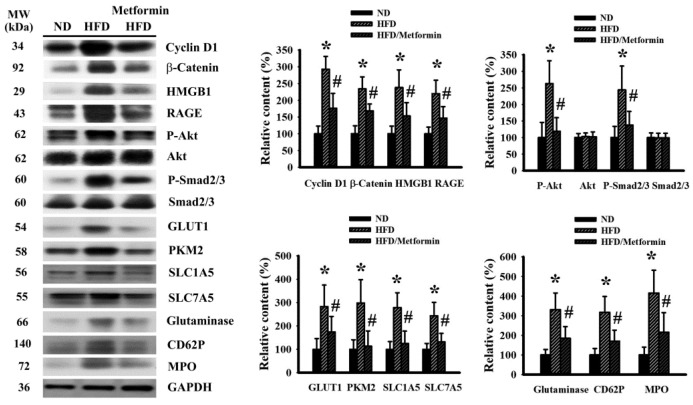
Metformin mitigated signaling molecule expression in tumor tissues. ND-fed lean and HFD-fed obese mice were subcutaneously inoculated with LLC cells over a course of three weeks. Three days after cell implantation, ND-fed lean mice were treated daily with saline vehicle through intraperitoneal injection (ND). HFD-fed obese mice were separated into two groups receiving daily intraperitoneal injections of a saline vehicle (HFD) or metformin (50 mg/kg, HFD/Metformin) for the final 18 days. The levels of specific proteins in the resected tumor tissues were measured using Western blot with indicated antibodies. Intensity of the specific protein was first normalized with that of GAPDH. For the calculation of relative content, the levels of normalized intensity in ND group were defined as 100%. Representative blots and quantitative data are shown. * *p* < 0.05 vs. ND and # *p* < 0.05 vs. HFD, n = 6.

To correlate with malignant progression and systemic alterations, changes in malignance-associated signaling molecules were examined from resected tumor tissues. Tumors seen in HFD-fed obese mice had an apparent increase in proliferation-promoting cycline D1, β-catenin, HMGB1, Receptor for Advanced Glycation End-Products (RAGE), Akt phosphorylation, Smad2/3 phosphorylation, aerobic glycolysis-associated Glucose Transporter-1 (GLUT1) and Pyruvate Kinase M2 (PKM2), glutaminolysis-associated Solute Carrier Family 1 Member 5 (SLC1A5), SLC7A5, glutaminase, platelet-associated CD62P and neutrophil-associated Myeloperoxidase (MPO). Those elevations were all mitigated by metformin (Figure 4). Therefore, we can surmise that obesity-driven cancer aggressiveness is accompanied by activations of proliferation, glycolysis, glutaminolysis, and platelet and neutrophil tumor infiltration, with the biochemical alterations being targeted by metformin.

### 2.3. Metformin Decreased LLC Cell Viability and Migration

**Figure 5:**
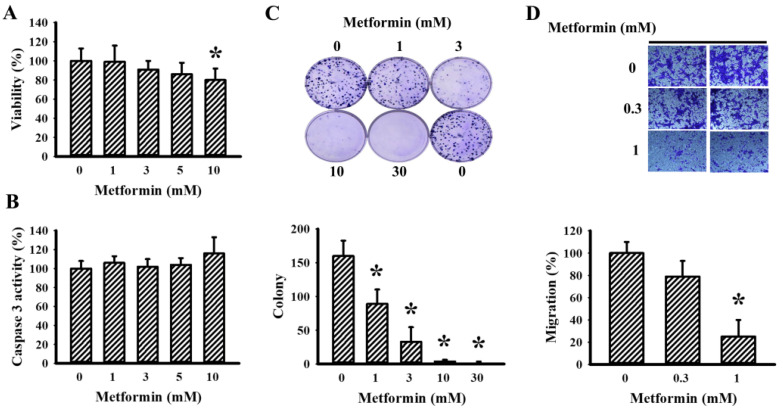
Metformin decreased LLC cell proliferation. Metformin (0–10 mM) was added to the cultured media of LLC cells for 24 h. Cell viability was measured using a CCK-8 kit (**A**) and caspase 3 activity was measured using an enzymatic assay kit (**B**). Metformin (0–30 mM) was added to the cultured media of LLC cells for 6 days. Cell colonies were visualized using crystal violet staining. Representative images and quantitative data are shown (**C**). Metformin (0–1 mM) was added to the cultured media of LLC cells, and cell migration within a course of 24 h was measured using a Transwell-based method. Representative photomicrograph and representative data are shown (**D**). * *p* < 0.05 vs. metformin (0 mM), n = 4.

Beyond the tumor growth inhibitory evaluation in tumor-bearing mice, tumor cellular responses to metformin were further examined in vitro. Metformin caused a slight reduction in cell viability when exposed to higher concentrations (Figure 5A), while having little effect on caspase 3 activity within the tested concentrations (Figure 5B). Additionally, lower concentrations of metformin were effective in decreasing both long-term cell growth (Figure 5C) and cell migration (Figure 5D). These results suggest that metformin has an inhibitory effect on cell proliferation and migration.

### 2.4. Metformin Decreased Signaling Molecule Expression in LLC Cells

**Figure 6:**
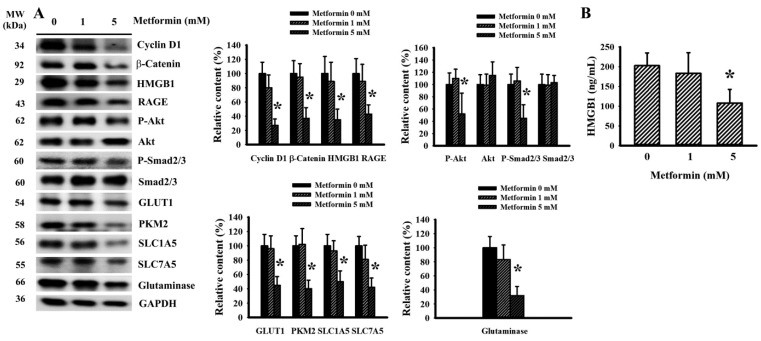
Metformin decreased signaling molecule expression in LLC cells. Metformin (0–5 mM) was added to the cultured media of LLC cells for 24 h. The levels of specific proteins in the cell lysates were measured using Western blot with indicated antibodies. Intensity of the specific protein was first normalized with that of GAPDH. For the calculation of relative content, the levels of normalized intensity in metformin (0 mM) group were defined as 100%. Representative blots and quantitative data are shown (**A**). The levels of HMGB1 in the cultured supernatants were measured using ELISA (**B**). * *p* < 0.05 vs. metformin (0 mM), n = 4.

In order to uncover any anticancer actions occurring due to metformin, proliferation and metabolism relevant signaling molecules were further examined in LLC cells. Decreased levels of cyclin D1, β-catenin, HMGB1, RAGE, Akt phosphorylation, Smad2/3 phosphorylation, GLUT1, PKM2, SLC1A5, SLC7A5 and glutaminase were revealed in cells treated with metformin (Figure 6A). Moreover, there was a reduction in the extracellular release of HMGB1 under metformin treatment (Figure 6B). Data taken from cell studies reveal that LLC cells respond to metformin treatment by decreasing the expression of proliferation-, aerobic glycolysis- and glutaminolysis-associated signaling molecules.

### 2.5. Metformin Mitigated Parameters of NETs in Tumor-Bearing Mice

**Figure 7:**
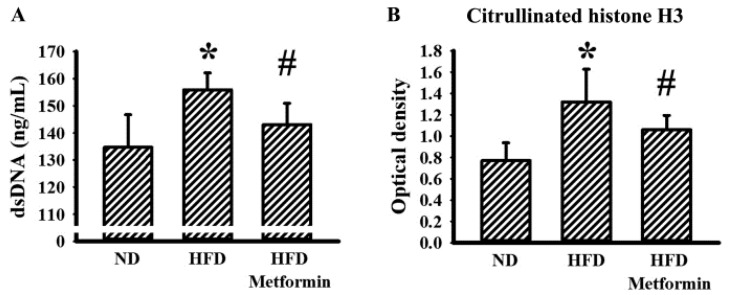
Metformin mitigated circulating parameters of NETs in obese mice. ND-fed lean and HFD-fed obese mice were subcutaneously inoculated with LLC cells over a course of three weeks. Three days after cell implantation, ND-fed lean mice were daily treated with saline vehicle through intraperitoneal injection (ND). HFD-fed obese mice were separated into two groups receiving daily intraperitoneal injections of a saline vehicle (HFD) or metformin (50 mg/kg, HFD/Metformin) for the final 18 days. The levels of dsDNA (**A**) and citrullinated histone H3 (**B**) in blood samples were measured, as described in methodology. * *p* < 0.05 vs. ND and # *p* < 0.05 vs. HFD, n = 6.

**Figure 8:**
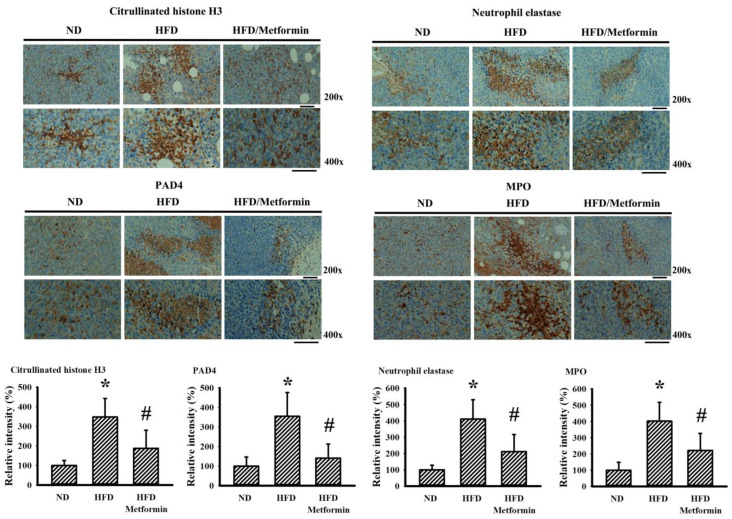
Metformin mitigated tissue parameters of NETs in obese mice. ND-fed lean and HFD-fed obese mice were subcutaneously inoculated with LLC cells over a course of three weeks. Three days after cell implantation, ND-fed lean mice were daily treated with saline vehicle through intraperitoneal injection (ND). HFD-fed obese mice were separated into two groups receiving daily intraperitoneal injections of a saline vehicle (HFD) or metformin (50 mg/kg, HFD/Metformin) for the final 18 days. Immunohistochemical examination was performed in paraffin-embedded tumor tissues with indicated antibodies. Representative photomicrograph and quantitative data are shown, as described in methodology. * *p* < 0.05 vs. ND and # *p* < 0.05 vs. HFD, n = 6. Scale bar: 50 μm.

Neutrophils and neutrophil-derived NETs have each been implicated in the aggressiveness of malignancy [8,25]. To explore any potential involvement of NETs in the tumor growth inhibitory effects of metformin, the parameters of NETs were examined in both the bloodstream and tumor tissues of tumor-bearing mice. Circulating levels of double-strandedDNA (dsDNA) (Figure 7A) and citrullinated histone H3 (Figure 7B) were slightly elevated in the blood samples taken from HFD-fed obese mice, with reductions later found after metformin treatment. Parallel changes were further demonstrated in the immunohistochemical detection of NET-associated citrullinated histone H3, neutrophil elastase, Protein Arginine Deiminase 4 (PAD4) and MPO in resected tumor tissues (Figure 8). Data taken from biochemical examinations show an association between systemic and tumor presence of NETs, and tumor aggressiveness in HFD-fed obese mice, suggesting a suppressive effect resulting from metformin.

### 2.6. Metformin Decreased NETs Induction In Vitro

**Figure 9:**
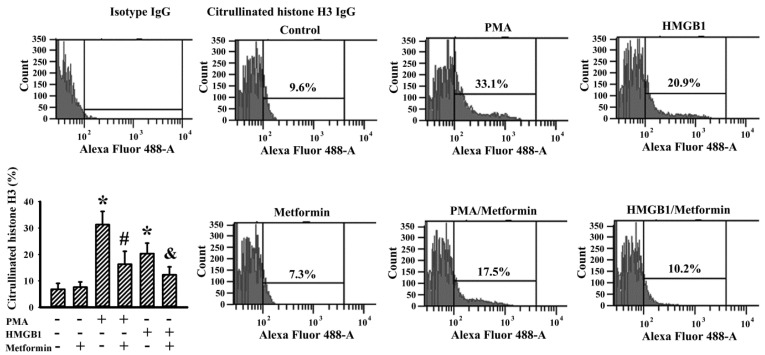
Metformin decreased NETs induction in human neutrophils. Human neutrophils were treated with a vehicle (Control), PMA (100 nM), HMGB1 (1 μg/mL), metformin (500 μM) or in combinations as indicated for 3 h. The treated neutrophils were subjected to flowcytometric analysis for the measurement of citrullinated histone H3 positivity with an indicated primary antibody and an Alexa Fluor 488-labeled secondary antibody. Control isotype IgG was used for the purpose of background calibration. Representative plots and quantitative data are shown. * *p* < 0.05 vs. untreated group, # *p* < 0.05 vs. PMA group, and & *p* < 0.05 vs. HMGB1 group, n = 3.

To gain further insight into the suppressive effects of metformin on NETs, in vitro human neutrophils were modeled for investigation. Similar to previous relevant studies [25,34,35,36], Phorbol Myristate Acetate (PMA) and recombinant HMGB1 effectively induced NET formation from human neutrophils, as evidenced by flowcytometric detection of citrullinated histone H3 immunopositivity (Figure 9). Metformin itself had a limited effect on spontaneous NET formation, although it decreased NET formation under PMA or HMGB1 stimulation (Figure 9). Data taken from a human neutrophils study suggest that there is a common inhibitory effect that metformin has on NET formation.

### 2.7. Cancer Cell Conditioned Medium Promoted PMA-Induced NETs

**Figure 10:**
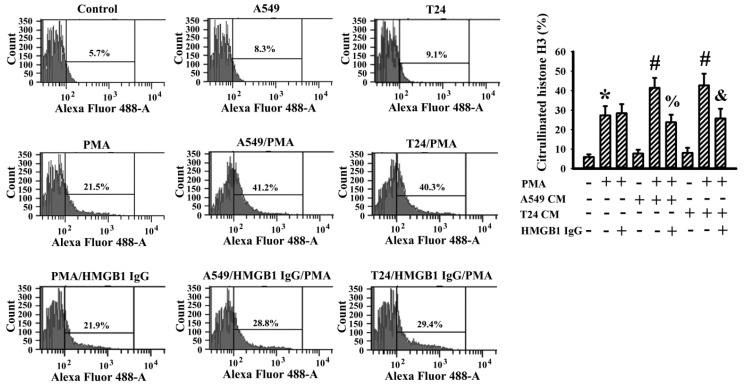
Conditioned medium of cancer cells promoted PMA-induced NETs in human neutrophils. Human neutrophils were treated with a vehicle (Control), PMA (100 nM) or PMA (100 nM) in combination with HMGB1-neutralizing IgG (5 μg/mL) for 3 h. For the study of cancer cell effects, supernatants of subconfluent A549 and T24 cells over a period of 24 h were collected as Conditioned Medium (CM). Human neutrophils were treated with A549 CM (with an equal volume of fresh medium), T24 CM (with an equal volume of fresh medium), or in combinations as indicated for 3 h. For the neutralization study, A549 CM and T24 CM were incubated with HMGB1-neutralizing IgG (5 μg/mL) for 15 min prior to being added to the neutrophils. The treated neutrophils were subjected to flowcytometric analysis for the measurement of citrullinated histone H3 positivity with an indicated primary antibody and an Alexa Fluor 488-labeled secondary antibody. Representative plots and quantitative data are shown. * *p* < 0.05 vs. untreated group, # *p* < 0.05 vs. PMA group, % *p* < 0.05 vs. PMA/A549 CM group, and & *p* < 0.05 vs. PMA/T24 CM group, n = 3.

To determine the presence of NETs in the resected tumor tissues, a crosstalk between cancer cells and neutrophils was investigated in an in vitro cell model. Human bladder cancer cell line T24 and human lung carcinoma cell line A549 were both cultivated, with the cultured conditioned medium then harvested for evaluation. Based on flowcytometric detection of citrullinated histone H3, conditioned medium of the A549 and T24 cells was relatively inert to the induction of NET formation from human neutrophils. However, both conditioned media promoted additional NET formation in responding to PMA stimulation (Figure 10). Although the presence of HMGB1-neutralizing IgG had a negligible effect on PMA-induced NET formation, the depletion of HMGB1 in an A549 and T24 conditioned medium through the neutralizing of IgG decreased their abilities to promote PMA-induced NET formation (Figure 10). To summarize, A549 and T24 cancer cells may accelerate or promote the ongoing NET formation in a cellcontact-independent mechanism, while extracellular released HMGB1 could be the candidate responsible for such an effect.

## 3. Discussion

Although the cancer-preventive effects of metformin remain controversial, its usage and subsequent reduction of cancer risk has been seen in certain cancer patients, particularly those subjects possessing a diabetic background [13,15,37,38]. To simulate clinical situations involving metformin prescription and use, experimental models of tumor growth in rodents have yielded promising evidence showcasing the anticancer potential of metformin [27,28,29,30,31]. Taking advantage of the syngeneic tumor models established by murine LLC cells and C57BL/6 mice, the parallel anticancer effects of metformin were demonstrated in this study when it centered on HFD-fed obese mice. HFD-fed obese mice displayed metabolic and pro-inflammatory alterations, with accompanying tumor aggressive growth, when compared with ND-fed lean mice. Metformin mitigated tumor growth in HFD-fed obese mice, and the tumor growth inhibition was associated with reductions in body mass, hyperglycemia, hyperinsulinemia, insulin resistance, WBCs, lymphocytes, neutrophils, plasma levels of leptin, soluble P-selectin, TGF-β1 and HMGB1, as well as the tumor levels of cyclin D1, β-catenin, HMGB1, RAGE, Akt phosphorylation, Smad2/3 phosphorylation, GLUT1, PKM2, SLC1A5, SLC7A5, glutaminase, CD62P and MPO, while there was an increase in plasma levels of glutamine. The suppressive effects of metformin on cell proliferation, migration and oncogenic signaling molecules were duplicated in an in vitro LLC cell study. Beyond those metabolic, pro-inflammatory and oncogenic changes, tumor-bearing HFD-fed obese mice displayed higher contents of circulating dsDNA and citrullinated histone H3, as well as tumor immunopositivity of citrullinated histone H3, neutrophil elastase, PAD4 and MPO. Based on in vitro models of human neutrophils, metformin exhibited inhibitory effects on PMA- and HMGB1-induced NET formation. Moreover, disruption of the interplay between cancer cells and neutrophils towards NET formation involving cancer cell-derived HMGB1 was found via a human A549 and T24 cancer cell study. Therefore, metabolic, pro-inflammatory and NET alterations appear to play pivotal roles in the obesity-driven aggressiveness of cancer, while also representing candidate targets for metformin with regard to anticancer treatment.

Beyond acting as a metabolic integrator, insulin coordinates cancer cell metabolism, proliferation and motility through its action on Akt/mTOR axis, lipogenesis and mitochondria. Therefore, both insulin and AMPK are two well-reported targets for the anticancer actions of metformin [16,17]. Cancer cells are highly proliferative and motile. To achieve such dynamically active cell behavior, a unique metabolic strategy coupling aerobic glycolysis and glutaminolysis is common and crucial to cancer cells. Insulin-independent GLUT1 moves glucose intracellular influx and PKM2 performs a quick catabolism of glucose, both of which are two key steps for aerobic glycolysis to fuel cancer cells with large amounts of ATP [39,40]. Meanwhile, glutamine influx through SLC1A5 and SLC7A5 glutamine transporters and subsequent glutaminase-mediated glutamate conversion provide a metabolic intermediate to boost the metabolic completion of the citric acid cycle, pentose phosphate pathway and glutathione synthesis [41]. In our previous study, we observed that LLC cancer cell implantation into ND-fed lean C57BL/6 mice appeared to have a negligible effect on circulating levels of glucose, insulin and leptin [32]. In this study, HFD-fed obese mice developed malignancy-prone metabolic environments, including hyperglycemia, hyperinsulinemia, insulin resistance, hyperleptinemia and elevated circulating glutamine. The corresponding tumors in the HFD-fed obese mice grew aggressively and expressed higher levels of proliferation-associated cyclin D1, β-catenin, Akt phosphorylation, aerobic glycolysis-associated GLUT1 and PKM2, glutaminolysis-associated SLC1A5, SLC7A5 and glutaminase. The cumulative elevation of circulating glutamine in tumor-bearing HFD-fed obese mice undergoing metformin treatment implied an impairment in glutamine uptake and metabolism. Consistent with the previous findings that showed metformin targets glucose metabolism, glutamine metabolism and mitochondrial activity [21,22], data taken from tumor tissue examinations and in vitro LLC cell studies revealed a suppressive effect that metformin has on cancer cell proliferation, together with a concurrent reduction in aerobic glycolysis and glutaminolysis. Through both the gathering of relevant studies and our findings, metabolic effects centering on glucose, lipids and glutamine remain key components of the anticancer mechanisms of metformin.

Both obesity and cancer are chronic diseases involving low-grade inflammation. Lymphocytes, neutrophils, platelets, TGF-β1 and HMGB1 have all been implicated in both pathogenesis and obesity-driven cancer [7,9,42,43,44]. During LLC cell-derived tumor growth in ND-fed lean C57/BL 6 mice over a period of 2 weeks, there was no remarkable change in the numbers of WBCs, lymphocytes, neutrophils or platelets, while elevations in the plasma levels of soluble P-selectin and TGF-β1 were noted [32]. Herein, tumor growth between HFD-fed obese and ND-fed lean mice was closely related to elevated circulating WBCs, lymphocytes, neutrophils, plasma levels of soluble P-selectin, TGF-β1 and HMGB1, as well as increased tumor expressions of HMGB1, RAGE, Smad2/3 phosphorylation, CD62P and MPO. Those biochemical changes in the blood circulation and tumor tissues of HFD-fed obese mice were alleviated by metformin. A parallel reduction in HMGB1, RAGE, Smad2/3 phosphorylation and extracellular release of HMGB1 were also demonstrated in metformin-treated LLC cells in vitro. Similar changes in signaling molecules have been revealed due to the anticancer effects of antiplatelet drugs, such as aspirin and dipyridamole, as well as exosome release inhibitor GW4869 [32,33]. Therefore, platelet and neutrophil activation and tumor infiltration, TGF-β1/Smad2/3 signaling and HMGB1/RAGE signaling all play substantial roles in the obesity-driven aggressiveness of cancer, with each being a potential target for the anticancer actions of metformin.

Neutrophils are key component cells in cancer progression and metastasis, having either cancer-promotion or cancer-inhibition effects. Accumulating evidence has highlighted that circulating and infiltrating neutrophils are proposed to be key mediators in neoplastic transformation, cancer cell proliferation and metastasis, malignant progression, angiogenesis, matrix remodeling and tumor immunity through NET structure [34,45,46]. Under certain situations, neutrophils undergo morphological and biochemical changes characterized by net-like structures consisting of extracellular dsDNA, hypercitrullinated histone and granular proteins such as elastase and MPO. During the transition from neutrophils to NET structure, the PAD4-controlled chromatin decondensation step is vital to the whole process, with PMA, LPS, HMGB1, cytokines, PKC and NADPH oxidases acting as common upstream inducers and intermediate mediators [25,34,35,36,45,46]. Tumor growth in HFD-fed obese mice had higher circulating dsDNA and citrullinated histone H3, plasma HMGB1, tumor contents of HMGB1, citrullinated histone H3, neutrophil elastase, PAD4 and MPO when compared to tumors in ND-fed lean mice. Those NETs-associated biochemical changes in tumor-bearing HFD-fed obese mice were suppressed by metformin. Unfortunately, the transition from murine neutrophils to NETs structure was not successfully demonstrated in the current study due to the low efficiency in in vitro NET induction from murine neutrophils [34]. Alternatively, we have demonstrated that PMA and HMGB1 are capable of induction of NET structures from human neutrophils, as well as their reversal by metformin. Since metformin is reported to have a suppressive effect on NET induction through PKC and NADPH oxidase mechanisms [25], the current findings have further revealed that it possesses an inhibitory effect on NETs induction, as it decreased not only PMA- but also HMGB1-induced NET formation.

HMGB1, a nonhistone DNA-binding protein, possesses pleiotropic biological activities, including cell proliferation, autophagy, inflammation and NET formation [35,36,43,47]. Previously, we found that HMGB1/RAGE signaling is of importance in cancer cell proliferation, survival and escape from apoptosis, and that the cancer cells and platelets are representative sources of HMGB1 release [32,33]. In the current study, we further identified that human A549 and T24 cancer cells promoted PMA-induced NET formation from human neutrophils involving released HMGB1 due to the suppressive effect of HMGB1-neutralizing IgG. Although the exogenous addition of HMGB1 at higher concentrations (1 μg/mL) displayed NET induction potential, a conditioned medium of A549 or T24 cells alone was relatively inert in NET induction from human neutrophils. Doseeffects could be a cause of this, as there were lower concentrations of HMGB1 (50–200 ng/mL) detected in the obtained conditioned medium. Despite the incomplete identification, results from the current study suggest that cancer cells may actively participate in the processes of NET formation through their release of promoting molecules, with HMGB1 being one such candidate.

Metformin has displayed anticancer effects towards different types of cancer cells in an in vitro cell study, with Nrf2, YAP, HIF-1α, HMGB1, TGF-β1, glycolysis and glutamine metabolism being proposed action targets at a cellular level [14,18,19,20,21,22,23]. However, the in vivo anticancer effects of metformin vary and depend on the accompanying situations. For example, metformin displays remarkable tumor growth inhibition in tumor-bearing immunodeficiency mice with concurrent hypoglycemia [48,49]. The parallel tumor growth inhibitory effects of metformin are commonly demonstrated in tumor-bearing rodents with obese or diabetic backgrounds [27,28,29,30,31]. Algire et al. [27] found that metformin had an inhibitory effect on tumor growth in C57BL/6 mice fed with HFD, while a limited effect was observed in C57BL/6 mice fed with a normal control diet. Although metformin use and its ability to reduce cancer risk are found in cancer patients [13,15,37,38], the aforementioned controversy indicates that metformin may only offer benefits to certain cancer populations with remarkable metabolic abnormalities.

Despite the current anticancer findings, there are limitations in the interpretation of the mechanistic actions of metformin. First, the effects of metformin on tumor growth without including any diet variables were not investigated. To have a better understanding of metformin’s anticancer actions and its application, a study involving a comparable group consisting of tumor-bearing mice that have been fed with a normal control diet and supplemented with metformin is of importance. Second, the metabolic actions may be a prerequisite to the anticancer effects of metformin. It should be noted that the improvements may also be secondary to weight loss or tumor reduction. Lastly, although the current study identified that NETs could be an action target of metformin’s anticancer effects, the detailed mechanistic actions towards NETs inhibition were not characterized. A deeper investigation centered on NETs in both tumor-bearing mice and neutrophil cell models is encouraged.

To extend the anticancer study of metformin, as it is centered on certain metabolic situations, a syngeneic tumor model was established through the implantation of LLC cells in HFD-fed obese C57BL/6 mice. Metformin treatment mitigated tumor growth, with accompanying alleviations seen in body mass, fasting glucose, fasting insulin, insulin resistance, circulating WBCs, lymphocytes, neutrophils, plasma leptin, soluble P-selectin, TGF-β1 and HMGB1, as well as the tumor signaling molecules of cyclin D1, β-catenin, HMGB1, RAGE, Akt phosphorylation, Smad2/3 phosphorylation, GLUT1, PKM2, SLC1A5, SLC7A5, glutaminase, CD62P and MPO, while elevation occurred in circulating glutamine. In vitro, metformin inhibited LLC cell proliferation and migration, while decreasing parallel oncogenic signaling molecules. Moreover, tumor-bearing HFD-fed obese mice displayed higher biochemical markers of the NET structure in the bloodstream and tumor tissues, with the suppressive effect being caused by metformin. Data taken from a human neutrophil study confirmed the inhibitory effect that metformin has on NET formation. Furthermore, HMGB1 was identified as a promoting molecule coming from cancer cells to boost the transition process toward NETs. Despite these remaining limitations, our current study has revealed that metabolic, pro-inflammatory and NET alterations appear to play pivotal roles in the obesity-driven aggressiveness of cancer, and represent candidate targets for metformin with regard to its anticancer abilities.

## 4. Materials and Methods

### 4.1. Cell Cultures

Human bladder carcinoma T24 (#60062), human lung carcinoma A549 (#60074) and murine LLC (#60050) cells were obtained from the Bioresource Collection and Research Center (Hsinchu, Taiwan). Upon propagation, T24, A549 and LLC cells were maintained in McCoy’s 5a medium, Ham’s F12K medium and Dulbecco’s Modified Eagle Medium (DMEM), respectively, with additions of 1% nonessential amino acids and 10% fetal bovine serum (FBS) in a 5% CO_2_ incubator at 37 °C. For conducting experiments, cells were placed in their corresponding media containing 5% FBS. Cell viability was evaluated using a Cell Counting Kit-8 (CCK-8, MedChemExpress, Monmouth Junction, NJ, USA), according to the manufacturer’s instructions.

### 4.2. Tumor-Bearing Mouse Model

A syngeneic tumor model was created through the implantation of LLC cells in C57BL/6 mice in accordance with our reported protocols [32,33]. The performance of experiments adhered strictly to the recommended guidelines and was approved by the Animal Experimental Committee of Taichung Veterans General Hospital (IACUC approval code: La-1101769, IACUC approval date: 10 February 2021). Male C57BL/6 mice (age 8 weeks) were separated into two groups, with one group being fed ND (10% energy from fat, 58Y2, TestDiet, St. Louis, MO, USA) and the other HFD (60% energy from fat, 58Y1, TestDiet, St. Louis, MO, USA) for 10 weeks. Under anesthesia using isoflurane, LLC cells (5 × 10^3^ cells in 100 μL of serum-free DMEM) were implanted subcutaneously into the right flanks of the mice. Three days after cell implantation, the ND group (n = 6) and half of the HFD group (n = 6) were intraperitoneally administered daily doses of normal saline, while the remaining half of the HFD group (n = 6) received an intraperitoneal metformin (50 mg/kg) injection. All mice were continuously fed their appropriate ND or HFD for an additional 18 days. The dosages and administration routes of metformin were according to the previous study [28]. After completion of the treatment course, the mice were euthanized and tumor tissues and blood samples collected for further analyses. The formula “V = (L × W^2^)/2 (L = length; W = width)” was applied for the calculation of tumor volume, and used as the reported method [32,33].

### 4.3. Blood Sample Collection and Analyses

Prior to being sacrificed, the mice were not allowed access to any food for 8 h. Under anesthesia with isoflurane, blood was withdrawn from the left femoral artery. WBCs, lymphocytes and neutrophils were each measured using routine complete blood counts. Plasma levels of insulin (#630-07289, *Shibayagi*, Gunma, Japan), TGF-β1 (DY1679-05, R&D Systems, Minneapolis, MN, USA), leptin (MOB00B, R&D Systems, Minneapolis, MN, USA), soluble P-selectin (DY737, R&D Systems, Minneapolis, MN, USA), glutamine (ab197011, Abcam, Cambridge, MA, USA) and HMGB1 (E4864, BioVision, Mountain View, CA, USA) were all measured using Enzyme Immunosorbent Assay (ELISA) kits, following the procedures provided by the respective manufacturers. Plasma levels of circulating citrullinated histone H3 were measured using self-prepared assay plates which had been coated with an antibody against citrullinated histone H3 (NB100-57135, Novus Biologicals, Centennial, CO, USA). The contents of immunocomplexes were quantified using a spectrophotometer at 450 nm. The level of fasting glucose was measured using a hand-held Accucheck glucometer (Roche Diagnostics, Indianapolis, IN, USA). The HOMA-IR index was calculated as [fasting insulin (μU/mL) × fasting glucose (mmol/L)]/22.5, and used as the reported method [32].

### 4.4. Western Blot

Whole steps of tissue protein extraction, cell lysate preparation, SDS-PAGE, Western blotting and quantitative calculation were performed as reported procedures [32,33]. Interesting molecules corresponding to the indicated antibodies were cyclin D1 (sc-56302), β-catenin (sc-133240), HMGB1 (sc-135809), RAGE (sc-74535), Akt (sc-5298), phospho-Akt (sc-293125), SLC7A5 (sc-374232), Smad2/3 (sc-398844), phospho-Smad2/3 (sc-11769) (Santa Cruz Biotechnology, Santa Cruz, CA, USA), GLUT1 (ab115730), PKM2 (ab137852), SLC1A5 (ab84903), glutaminase (ab150474), CD62P (ab59738), MPO (ab65871) (Abcam, Cambridge, MA, USA) and Glyceraldehyde-3-Phosphate Dehydrogenase (AF5718, GAPDH) (R&D Systems, Minneapolis, MN, USA).The proteins of interest were visualized and quantified using a G:BOX mini multi-fluorescence and chemiluminescence imaging system (Syngene, Frederick, MD, USA).

### 4.5. Clonogenic Assay

To perform clonogenic assay, LLC cells were seeded onto a 6-well plate at a density of 500 cells per well. The following day, cells began treatment using various concentrations of metformin (0–30 mM) in a DMEM, supplemented with 5% FBS over the course of 6 days. To visualize the grown cell colony, cells were stained with crystal violet.

### 4.6. Cell Migration Assay

Cell migration was evaluated using a method based on a 24-well Transwell apparatus according to reported procedures [33]. LLC cell suspensions (2 × 10^4^) in 200 μL DMEM containing 2% FBS with the presence of various concentrations of metformin (0–1 mM) were added to the Transwell inserts (8 μm pore size, BD Falcon Cell Culture insert, BD Biosciences, San Jose, CA, USA). The Transwell inserts were put into a 24-well plate and the lower chambers were filled with DMEM containing 10% FBS for a course of 24 h. Cells that trans-migrated to the lower surfaces of the Transwell inserts were stained with Giemsa. During visualization under a light microscope, six random fields per insert were picked up, with cell numbers then being counted for evaluation.

### 4.7. Measurement of Caspase 3 Activity

Caspase 3 activity in the resected tumor tissues was measured using a Caspase Fluorometric Assay Kit (BioVision, Mountain View, CA, USA) according to the manufacturer’s instructions. Equal amounts of extracts were subjected to the reaction, with the yielded fluorescence signals measured using a fluorometer (E_x_ 380 nm and E_m_ 460 nm).

### 4.8. Measurement of dsDNA

To measure the plasma levels of dsDNA, samples were incubated with Sytox Green (fluorescent dsDNA-binding dye, Thermo Fisher Scientific, Waltham, MA, USA) at a concentration of 1 μM for 5 min. The fluorescence signals were measured with a fluorometer (E_x_ 480 nm and E_m_ 520 nm) and calibrated with a standard curve.

### 4.9. Immunohistochemistry

Paraffin-embedded tumor tissues were allocated for immunohistochemical examination using the corresponding antibodies. Deparaffinized sections (5 μm) were incubated with anti-citrullinated histone H3 (NB100-57135, Novus Biologicals, Centennial, CO, USA), anti-neutrophil elastase (NBP2-66972, Novus Biologicals, Centennial, CO, USA), anti-PAD4 (ab96758, Abcam, Cambridge, MA, USA) and anti-MPO (ab65871, Abcam, Cambridge, MA, USA) antibodies. After further incubation with biotinylated secondary antibodies and avidin–biotin–peroxidase complex, sections were developed with diaminobenzidine and counterstained with hematoxylin. The immunoreactive intensity in the sections was then quantified using Image J software (National Institutes of Health, Bethesda, MD, USA).

### 4.10. Flow Cytometric Measurement of Citrullinated Histone H3

To determine NETs induction in vitro, human neutrophils were prepared from 10 healthy volunteers of both genders after obtaining written informed consent. The study protocol (IRB approval code: CE21217A, IRB approval date: 2 July 2021) was approved by the Taichung Veterans General Hospital Institutional Review Board. Peripheral blood (10 mL in an EDTA tube) was drawn from each individual participant and diluted with an equal volume of dextran 500 (3%). After red blood cell sedimentation, the upper layer was gently nestled on top of a Histopaque-1077 solution, followed by centrifugation at 400× *g* for 30 min. The high-density fraction pellets were then subjected to red blood cell lysis [34]. The obtained neutrophils (more than 95% purity) were maintained in an RPMI medium containing 0.5% FBS for further analyses. To obtain a Conditioned Medium (CM), A549 and T24 cells were allowed to grow subconfluently for 24 h. The cultured media were then collected and centrifuged at 1000× *g* for 10 min. The supernatants were named CM. For the induction of NETs, neutrophils were treated with a vehicle, either PMA (100 nM), HMGB1 (1 μg/mL), metformin (500 μM), A549 CM (with an equal volume of fresh medium), T24 CM (with an equal volume of fresh medium) or HMGB1-neutralizing IgG (5 μg/mL, ab77302, Abcam, Cambridge, MA, USA), or with combinations of vehicles for a period of 3 h. For the neutralization study, A549 CM and T24 CM were incubated with HMGB1-neutralizing IgG (5 μg/mL) for 15 min prior to being added to the neutrophils. After fixation and permeabilization, neutrophils were then incubated with either an anti-citrullinated histone H3 antibody or isotype IgG (NB100-57135, Novus Biologicals, Centennial, CO, USA), followed by an Alexa Fluor 488-labeled secondary antibody (Thermo Fisher Scientific, Waltham, MA, USA). The levels of citrullinated histone H3 immunopositivity were measured using the FACSCalibur and analyzed by Cell Quest Pro software (BD Biosciences, San Jose, CA, USA).

### 4.11. Statistical Analysis

Graph preparation and data analyses were performed with SigmaPlot (12.3) and GraphPad Prism 8.0 Software (2020, San Diego, CA, USA). For multiple groups and variables, one-way analysis of variance (ANOVA) with a Bonferroni or Dunnett post hoc test was performed. For the in vitro NETs study, data are representative of three biological replicates from each individual participant. All values are listed as mean values ± standard deviation (SD), with differences between groups being statistically significant at a *p* value less than 0.05.

## Data Availability

Not applicable.
